# Microstructural differences between naturally-deposited and laboratory beach sands

**DOI:** 10.1007/s10035-021-01169-4

**Published:** 2021-11-11

**Authors:** Amy Ferrick, Vanshan Wright, Michael Manga, Nicholas Sitar

**Affiliations:** 1 Department of Earth and Planetary Science, University of California Berkeley, Berkeley, California 94720 USA; 2 Geology and Geophysics Department, Woods Hole Oceanographic Institution, Woods Hole, Massachusetts 02543 USA; 3 Scripps Institution of Oceanography, University of California San Diego, La Jolla, California 92037 USA; 4 Department of Civil and Environmental Engineering, University of California Berkeley, Berkeley, California 94720 USA

**Keywords:** Microstructure, X-ray computed microtomography, Coordination number, Pluviation

## Abstract

The orientation of, and contacts between, grains of sand reflect the processes that deposit the sands. Grain orientation and contact geometry also influence mechanical properties. Quantifying and understanding sand microstructure thus provide an opportunity to understand depositional processes better and connect microstructure and macroscopic properties. Using x-ray computed microtomography, we compare the microstructure of naturally-deposited beach sands and laboratory sands created by air pluviation in which samples are formed by raining sand grains into a container. We find that naturally-deposited sands have a narrower distribution of coordination number (i.e., the number of grains in contact) and a broader distribution of grain orientations than pluviated sands. The naturally-deposited sand grains orient inclined to the horizontal, and the pluviated sand grains orient horizontally. We explain the microstructural differences between the two different depositional methods by flowing water at beaches that re-positions and reorients grains initially deposited in unstable grain configurations.

## Introduction

Sand deposits are formed by accumulation of individual grains. The transporting medium and sedimentary environment will influence sands’ microstructure, including the porosity, coordination number (i.e., the number of contacts between grains), spatial organization, and orientation of grains (e.g., [1]). These microstructural properties influence macroscopic properties of sands, including elastic properties and hence seismic velocities [2–4], strength and particle breakage [5, 6], and liquefaction susceptibility [7, 8]. Thus, microstructure presumably explains behavioral differences of sands deposited differently [9–11].

Experimental measurements of sands’ physical properties typically rely on samples reconstituted using different methods of sample preparation such as wet and dry tamping and wet and dry pluviation [12]. However, it has been well documented that reconstituted sands’ mechanical properties are a function of the sample preparation method and, therefore, they do not necessarily behave the same as in situ sands [13]. In natural beach deposition, depositional energy is relatively high, flowing water deposits grains, and swash and backwash continuously operate. In air pluviation, which involves raining dry sands from a certain height into a container, depositional energy is relatively low, gravity deposits grains in air, and (ideally) no post-depositional processes operate. Since the processes depositing the particles in natural deposition and pluviation are different, the microstructure and physical properties differ as well [14]. In pluviated sands, particles’ long axes preferentially orient in the horizontal plane (i.e., perpendicular to the local gravity direction) and symmetrically distribute around the vertical axis (i.e., parallel to the local gravity direction) [15]. Contact normals tend to orient vertically due to gravity [16]. Sands deposited in nature generally develop long axis orientations parallel to the moving medium’s direction (e.g., ocean water), although beach sands have more complicated orientations because the direction and magnitude of waves, swash, and backwash vary over time [17]. Little work has been done to describe contact geometry of undisturbed naturally-deposited sands. Despite different depositional processes, the average coordination number of pluviated sands is similar to that of naturally-deposited sands with the same porosity [18].

Previous studies have attempted to relate depositional method to microstructure (e.g., [1]), albeit using manual analysis methods. Thus, these studies are limited to a relatively small number of grains and are more conducive to qualitative rather than quantitative analysis. X-ray microtomography paired with image analysis methods permit large-scale quantification of microstructure, and thus improves on previous methods.

Because deposition influences microstructure, deposition may also influence the stability of grain configurations [1], as stability is determined by the relative positions of touching grains. Granular assemblies are often characterized as “jammed” if stable and “unjammed” if unstable [19]. Among other key parameters, such as porosity and shear stress, coordination number strongly influences degree of jamming; in three dimensions, and for spherical grains, a minimum mean coordination number of 6 is required for a jammed state [19]. Jamming and unjamming can be triggered by wedging and unwedging of rattlers (unjammed grains confined in a pore) [20].

Here, we aim to understand how naturally-deposited beach sand differs from reconstituted beach sand to better understand the influence of depositional processes on microstructure. We investigate the effect of depositional history by comparing the microstructures of pluviated and naturally-deposited samples of the same sand. We use x-ray computed microtomography to reconstruct 3-D volumes of pluviated and naturally-deposited beach sand from Alameda County, California. We use image analysis techniques to quantify microstructural properties, including porosity, coordination number, grain orientation, and contact normal orientation. We find that the distributions of microstructural properties differ for the two depositional methods.

## Methods

To compare the microstructures of naturally-deposited and pluviated sands, we first collected sand cores from a natural beach. We then pluviated a sample with sand from the same beach. We acquired three-dimensional x-ray computed microtomographic images of the samples. Image analyses, followed by statistical analyses, allowed us to quantify and compare the microstructures of the sands deposited by the two deposition methods.

### Sample collection

We collected three undisturbed samples of naturally-deposited sand from an unnamed beach in Alameda County, California, USA (37$^\circ$51’04” N, 122$^\circ$18’00” W). Collection took place at low tide, approximately 7 m from the waterline, and at depths of 1 cm, 6 cm, and 11 cm. We measure depth as the vertical distance below the local surface. These shallow depths were chosen because sands are initially deposited at a depth of 0, and thus shallow sands should be most relevant for understanding depositional processes. Further, sands at depths greater than 15 cm were fully saturated, preventing sample collection. Our sample collection technique has been previously successfully implemented by Sitar et al. [14]. We collected the samples by gently inserting a transparent plastic straw into the sand at each depth. The straws are 11 mm in diameter and 22 mm in length. After inserting the straws into the sand, we then removed the sand around the outside of the straw before gently removing the straw. Before transporting the straws, we temporarily sealed the straws with tape and wrapped the straws in paper towels for moisture insulation. To ensure preservation of the samples, we then covered each end of the samples with cheesecloth and enclosed the entire straw with melted wax.

To create the pluviated sample, we poured dried sand from the Alameda County collection site through an 11 mm-diameter funnel opening held 30 cm above a plastic straw. We chose this height to achieve a similar porosity to that of the naturally-deposited samples [12]. We sealed the pluviated sample with cheesecloth and wax in a similar fashion.

### XRCT imaging

We acquired x-ray computed microtomography images of each sample on beamline 8.3.2 at the Advanced Light Source, Lawrence Berkeley National Lab. We imaged using 30 keV monochromatic x-rays, a 200-millisecond exposure time, and collected 1969 projections during continuous sample rotation through 180°. Each image volume comprises  500 two-dimensional image slices. We captured the images using a PCO edge camera, a 1X Nikon lens, and a 50 mm LuAG scintillator. The linear dimension of each voxel is 6.45 $\mu$m. We used Xi-cam software for image reconstruction [21], including center of rotation optimizations (correct determination of the axis the sample is rotated about), ring removal (correction of rings of erroneous pixel values centered about the rotation axis), and outlier removal (correction of local erroneous pixels).

### Image analyses

Image analysis allowed us to identify individual grains and quantify their properties. We first binarized the images (i.e., separated each voxel into the ‘grain’ phase or ‘pore’ phase) using ImageJ’s machine learning algorithm, Trainable Weka Segmentation [22, 23]. This machine learning segmentation method incorporates user knowledge and is thus well-suited to image data for which traditional segmentation methods fail [23]. The algorithm uses user input (e.g., manually segmented phases) to learn pixel classification. We train the classifier on the original image, as well as one image with each user-selected training feature applied (e.g., edge detection filters, texture filters). We trained the classifier with approximately five manually segmented two-dimensional grains and pores on every 50$^{\mathrm {th}}$ two-dimensional vertical image slice in the image volume representing each sample. We chose Gaussian blur as the training feature because, upon testing various training features on our image data, Gaussian blur produces a segmentation that best represents the grains and pores visible in the original grayscale images. The classifier is trained on the original images and blurred versions, each with a different Gaussian sigma value (minimum sigma = 1, maximum sigma = 8). The Gaussian sigma, which is the standard deviation of the underlying Gaussian distribution, determines how blurred the images are. We manually inspected the binarization quality by visually comparing the binarized image volume with the original image volume.

Individual grains must be identified from the binarized image volume. To this end, we identified and labeled each grain using the 3D Distance Transform Watershed [24], which uses a distance map (i.e., the set of weights used to approximate Euclidean distance) to calculate distances from objects’ centers. Object borders are then placed by maximizing the distance between touching objects’ centers. We chose the Borgefors distance map because it best approximates Euclidean distance [25]. The Borgefors distance map assigns distances of 3 to voxels sharing a face, 4 to voxels sharing an edge, and 5 to voxels sharing a point. The distance transform watershed also takes in a dynamic parameter, which influences the degree of segmentation, and a voxel connectivity parameter, which influences object roundness. Upon testing different parameters, a dynamic parameter of 2 and a connectivity parameter of 6 produce the most visually accurate segmentation of our image data. The segmentation removes one pixel-wide gap between touching grains, so we applied a morphological closing filter using a ball structuring element to reestablish contacts. Finally, we applied image multiplication with the binary image to remove any errors introduced by the morphological closing. To quantify precision of the segmentation process, we performed this process on a 15-image subset of one of the samples 15 times (see Sect. 3.1).

We used Software for Practical Analysis of Materials [26] to quantify each sample’s microstructural properties, including porosity, coordination number, contact normal orientation, and grain orientation. In a binary cylindrical subvolume, porosity is measured as the ratio of “pore” voxels to total voxels. We computed grain surface area using a discretization of the Crofton formula [24]. To quantify fabric anisotropy (i.e., the directional variation of particle arrangements), we used a scalar anisotropy factor defined as

1 $$\begin{aligned} a=\frac{15}{2}\sqrt{\frac{3}{2}R_{ij}^{'} R_{ij}^{'}}, \end{aligned}$$

where $R_{ij}^{'}$ is the deviatoric part of the grain orientations’ fabric tensor [27]. The fabric tensor characterizes the directional distribution of orientations and is defined in [28]. Fabric parameters are computed on grain orientations, denoted by the subscript *G*, and contact normal orientations, denoted by the subscript *CN*. In order to improve the accuracy of the contact orientation calculations, we first apply a random walker to further segment contacting grains. For a unimodal grain size distribution, grain size sorting is calculated as the Inclusive Graphic Standard Deviation [29]:

2 $$\begin{aligned} \sigma _I=\frac{\phi 84-\phi 16}{4}+\frac{\phi 95-\phi 5}{6.6}, \end{aligned}$$

where $\phi 84$ is the phi value of the 84th percentile of grain size distribution. The phi value of a given grain diameter is

3 $$\begin{aligned} \phi =-\log _2D, \end{aligned}$$

where *D* is the grain diameter in mm. Sphericity is calculated as

4 $$\begin{aligned} S=\frac{32\pi V^2}{A^3}, \end{aligned}$$

where *V* is grain volume and *A* is grain surface area [26].

Finally, we performed a t-test and a Kolmogorov-Smirnov test to compare microstructural properties’ distributions for the two depositional methods. The test statistic for the t-test is the difference between the parameter means, and the test statistic for the Kolmogorov-Smirnov test is the maximum difference between the parameter cumulative distribution functions. As is conventional in statistical hypothesis testing, low P-values indicate that differences in the data are significant.

**Table 1:** Microstructural properties of the sands

Deposition	Depth (cm)	N$^a$	$\phi ^b$	Mean coordination number ($\pm 1\sigma$)	$a_G$	$a_{CN}$	Mean grain volume (mm$^3$)	Mean grain surface area (mm$^2$)	Mean grain diameter (mm)	$\sigma _I$	Mean sphericity
Natural	1	22985	0.403	8.15±3.64	0.38	0.16	0.0070	0.21	0.22	0.50	0.82
Natural	6	12521	0.374	7.71±3.01	0.34	0.14	0.0122	0.30	0.26	0.48	0.82
Natural	11	18458	0.371	8.31±3.36	0.41	0.27	0.0078	0.23	0.23	0.48	0.82
Pluviated	n/a	24103	0.385	7.45±3.64	0.53	0.42	0.0051	0.16	0.19	0.55	0.83

$^a$Number of grains

$$^b$$Porosity

**Fig. 1:**
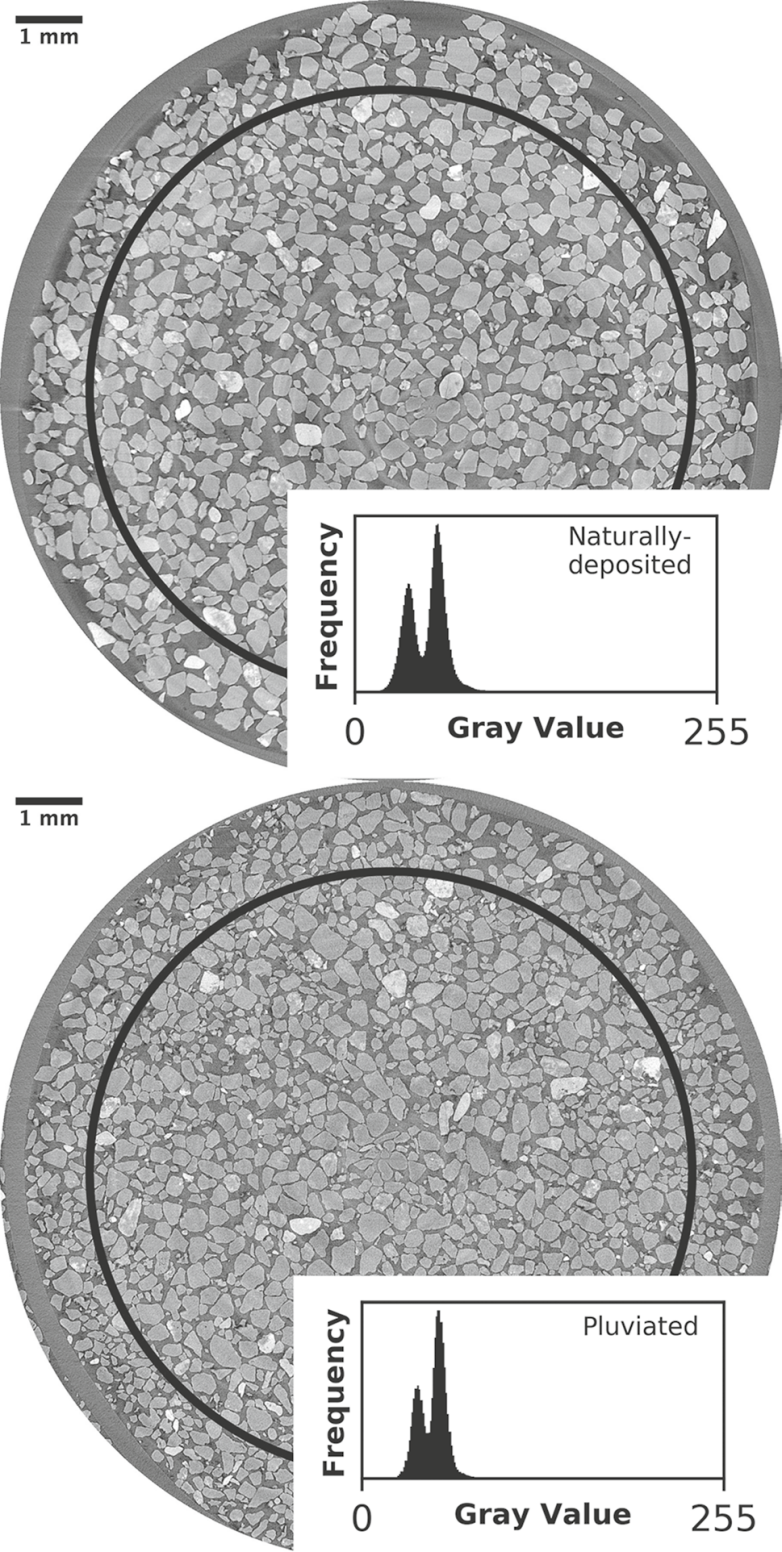
X-ray computed microtomography images showing horizontal cross-sections of the naturally-deposited (top) and pluviated (bottom) sands. The black circles denote the 9 mm diameter of the image subsection we consider in our analyses

## Results

We find that (1) x-ray computed microtomography data retain the microstructures of the sands, (2) grain and pore properties can be reliably compared for images of the same resolution, and (3) the distributions of coordination numbers and grain orientations in pluviated and naturally-deposited sands differ.

### Uncertainty and resolution

The x-ray microtomography data capture the microstructural properties of the sands. Grain and pore distributions appear consistent within the inner 9 mm of each sample (Fig. 1). Some anomalously large pores exist within 1 mm of the sample walls, suggesting that the microstructure was disturbed immediately adjacent to the sampling tube. Indeed, porosity within 1 mm of the sample boundaries is 14% greater than porosity in the rest of the sample, which varies by up to only 3%. Thus, we only analyze the innermost 9 mm of the samples.

Our segmentation procedure produces consistent results. When we segment a 15-image subset 15 different times, the estimated porosities differ by 2%. Repeating the segmentation process on an entire sample 3 different times results in the following variation: 3.4% in porosity, 4.9% in number of grains detected, 6.9% in mean coordination number, 4.1% in standard deviation of coordination number, 8.2% in mean grain surface area, 3.5% in standard deviation of grain surface area, and 4.0% in anisotropy of grain orientations. Thus, differences in results introduced by the segmentation procedures are small compared to the differences between pluviated and naturally-deposited sands that we interpret and discuss.

**Fig. 2:**
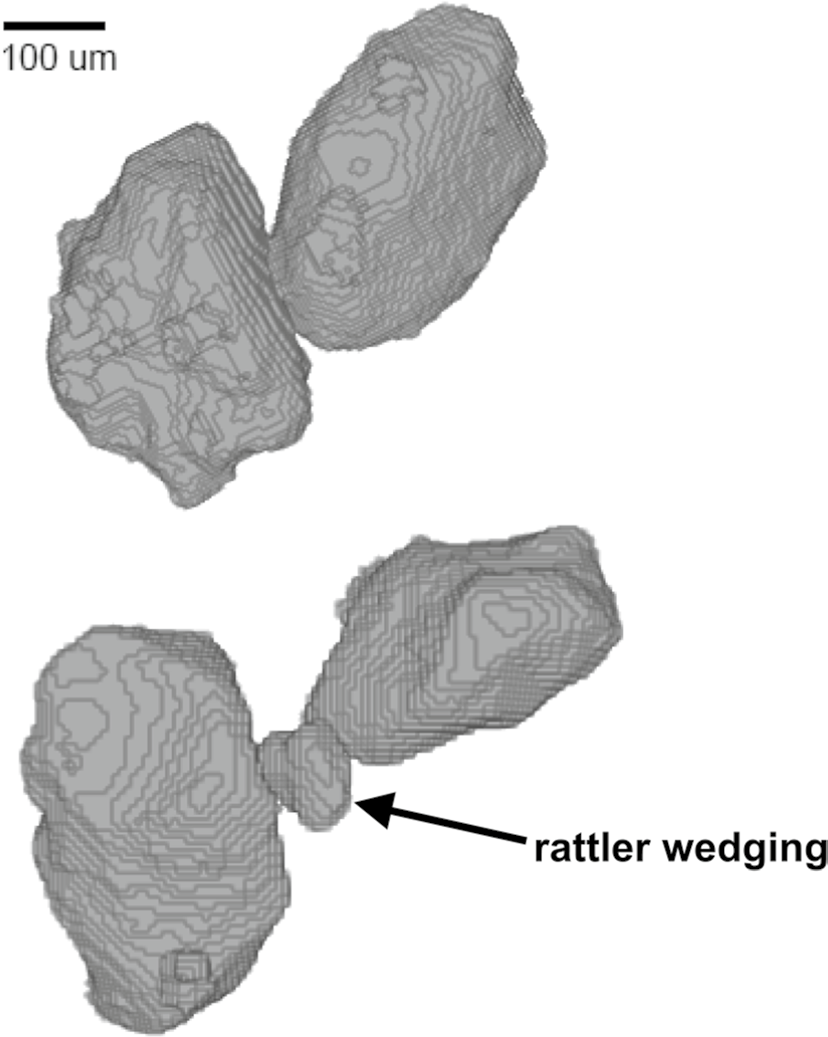
3-D rendering of two different grain configurations, including two large grains in contact (top), and a small grain wedged between two large grains (bottom)

**Table 2:** Results of T-test and Kolmogorov-Smirnov test

Test	Coordination number		Long axis elevation angle	
	Score	*P*-value	Score	*P*-value
T	7.25	4.24E-13	13.52	1.48E-40
K-S	0.11	2.09E-80	0.051	4.17E-36

A comparison between the x-ray microtomography images of pluviated and naturally-deposited sands is viable because the image volumes share fundamental characteristics. The images exhibit similar levels of resolution, noise, and blur (Fig. 1). The signal-to-noise ratio, which is computed using all voxels comprising a single phase, is the mean voxel intensity divided by the standard deviation voxel intensity. The pore phase signal-to-noise ratio is comparable in a naturally-deposited sample (6.57) and the pluviated sample (8.31). Further, the segmented image volumes capture grain contacts at a high resolution (Fig. 2). Images of finite resolution produce systematic, resolution-dependent overdetection of grain contacts [30, 31]. However, using identical scanning parameters and image processing techniques is expected to produce similar errors for sands with similar grain morphologies. Thus, even when both images have systematic uncertainties, comparing contact measurements can still identify differences between the pluviated and naturally-deposited sands.

**Fig. 3:**
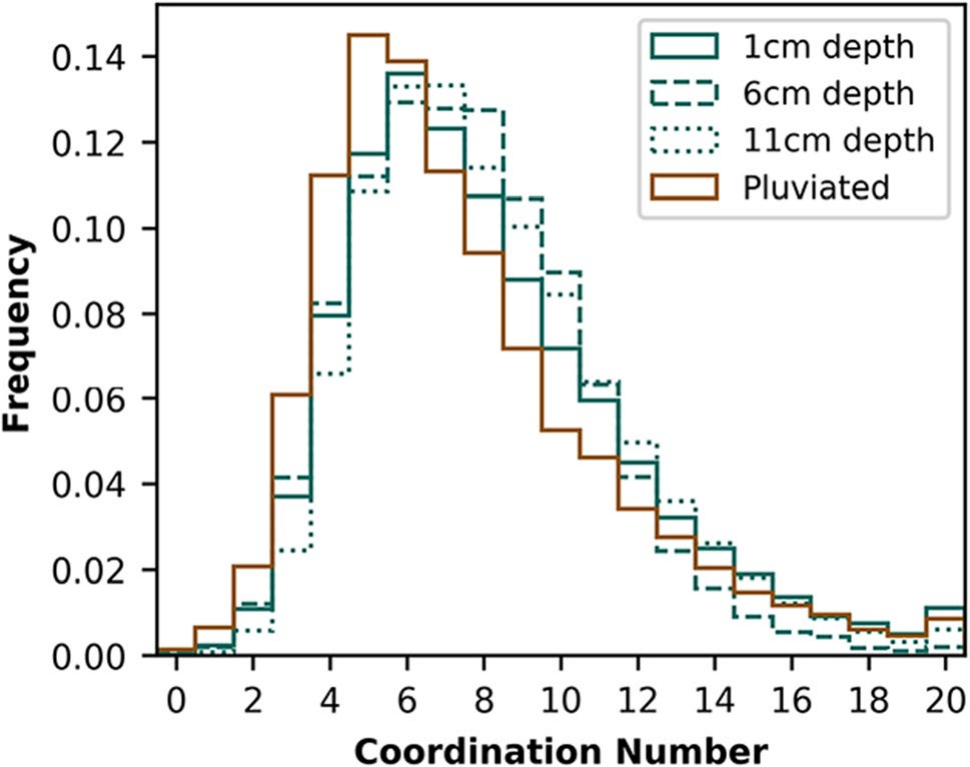
Distribution of coordination number for naturally-deposited and pluviated samples

**Fig. 4:**
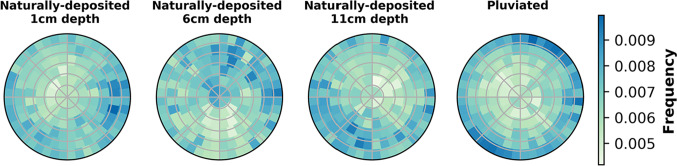
Distributions of grain long axis orientation on a Lambert azimuthal equal-area projection. Circular grid lines are at increments of 15$^\circ$

### Pluviated and naturally-deposited microstructure

The pluviated and naturally-deposited sands exhibit distinct microstructures (Table 1). The porosities of the naturally-deposited samples are 0.40, 0.37, and 0.37 from shallowest to deepest samples. The porosity of the pluviated sample is 0.38. Local porosity decreases by 10% in the uppermost 1 cm of the shallowest naturally-deposited sample. No other sample exhibits significant vertical variation in porosity. Mean coordination number is lower in the pluviated sands (7.45) than in the naturally-deposited sands (8.15, 7.71, and 8.31 from shallowest to deepest). Standard deviation for coordination number in the pluviated sands (3.66) is higher than the naturally-deposited sands (3.36, 3.01, and 3.36 from shallowest to deepest). Coordination numbers in all samples range from 2 to 20 (Fig. 3). The naturally-deposited sands have a lower frequency of grains with low coordination numbers (<5) and a lower frequency of grains with high coordination numbers (>14) than the pluviated sands (Fig. 3). All samples exhibit unimodal grain size distributions, and thus equation (2) is appropriate.

**Fig. 5:**
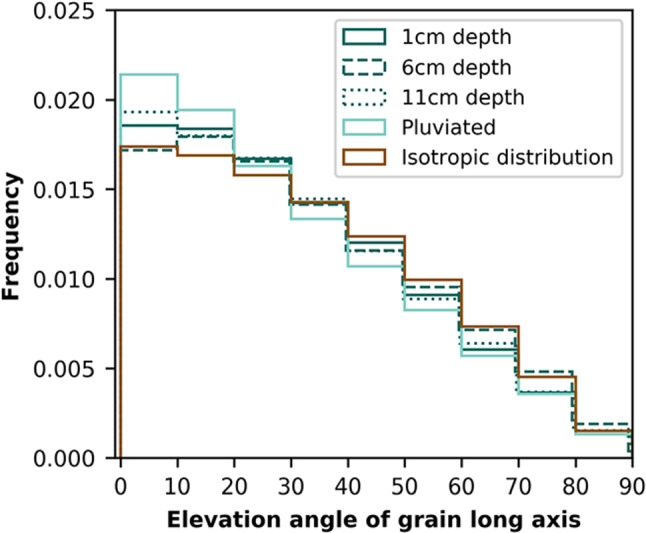
Distributions of elevation of grain long axis

Grain orientation is described with two angles: azimuth (i.e., the angle between North and the long axis projected onto the horizontal plane) and elevation (i.e., the vertical angle from the horizontal). Figure 4 presents three-dimensional histograms of grain long axis orientations on an equal-area spherical projection; vertically-oriented grains plot at the center, and horizontally-oriented grains plot along the circumference. Figure 5 shows the distributions of long axis elevation angles in each sample compared to a distribution of isotropically oriented grains. All samples exhibit a higher frequency of low elevation angles compared to the isotropic distribution (Fig. 5). Both the pluviated and naturally-deposited sands prefer elevations of 0 to 30 degrees from the horizontal (Figs. 4 and 5). This preference is markedly greater in the pluviated sands, especially for very small elevations (e.g., 0 to 10 degrees). The preferred azimuth in the naturally-deposited samples has a range of approximately 180 degrees (Fig. 4). The pluviated sands exhibit a higher degree of fabric anisotropy than the naturally-deposited sands (Table 1).

**Fig. 6:**
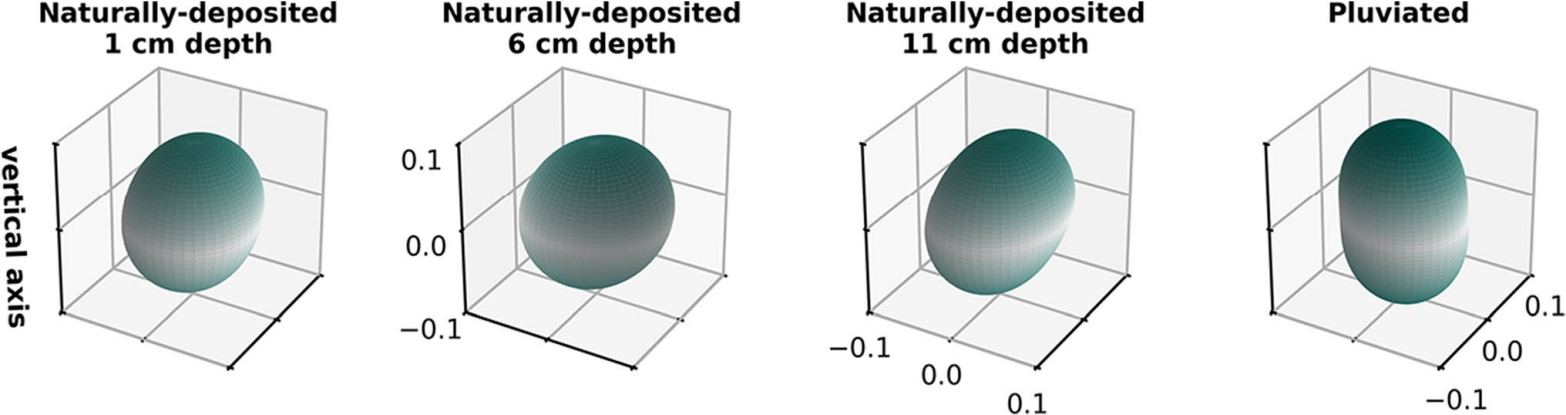
Distribution density of the fabric tensor computed from contact normal orientations. Axes represent the normalized distribution density in each of the three directions. Surfaces are colored by distance from 0 in the vertical direction

With respect to contact geometry, the pluviated sands are distinct from the naturally-deposited sands (Fig. 6). Contact normals mainly orient vertically in the pluviated sands (e.g., 0 to 15 degrees from the vertical). In all three naturally-deposited samples, contact normals tend to distribute at an angle (e.g., 30 to 60 degrees) from the vertical.

The means and distributions of coordination number and grain orientation in the naturally-deposited and pluviated samples are significantly different; p-values of both statistical tests are small (Table 2). The t-test and Kolmogorov-Smirnov test have 36,622 degrees of freedom for coordination number and 72,128 degrees of freedom for long axis elevation angle.

## Discussion

We identified two primary differences between natural and pluviated sands: (1) naturally-deposited sands have a lower frequency of grains with low (<6) and high (>16) coordination numbers, and (2) naturally-deposited sands, unlike pluviated sands, have an inclined preferred grain orientation. We now argue that perturbations from flowing water at beaches can explain these microstructural differences.

### Coordination number

Differences in coordination numbers can be explained by the effects of flowing water in naturally-deposited beach sands. Flowing water, such as swash and backwash on a beach, preferentially mobilizes small grains [32]. We propose that mobilization of small grains can explain infrequent low and high coordination numbers in naturally-deposited sands. Rattlers wedged between large grains (e.g., Fig. 2) may be mobilized by flowing water and allowed to find configurations with a higher number of contacts. Because rattlers, including those in wedged positions, have low coordination numbers (e.g., Fig. 2), this process may explain the depletion in low coordination numbers observed in naturally-deposited sands compared with pluviated sands (Fig. 3). Thus, flowing water may facilitate an increase in degree of jamming by reducing low coordination numbers. When a wedged rattler is removed, the two formerly separated large grains can contact each other. Contact with a large grain occupies more of a grain’s surrounding volume than contact with a small grain (see Fig. 2), limiting ability to contact other grains. Thus, flowing water can also explain the depletion in very high coordination numbers observed in naturally-deposited sands (Fig. 3). This interpretation is consistent with existing studies that found that sand columns created by air pluviation have a higher number of unstable grain configurations than sands formed by water sedimentation (e.g., Ref. [1]). These studies, while able to manually identify and count unstable and stable grains, consider fewer grains and do not constrain microstructure using x-ray microtomography.

All samples have a mean coordination number greater than 6, the minimum required for jamming for spherical grains [19]. Note that the critical coordination number for irregularly-shaped grains may be different than for spheres. Even if an entire assembly is stable, local instabilities, with local coordination number less than critical, may exist. All low coordination numbers (e.g., less than 6) are more frequent in the pluviated sands (Fig. 3), indicating a more weakly jammed state than that characterizing naturally-deposited grains.

### Grain orientation

**Fig. 7:**
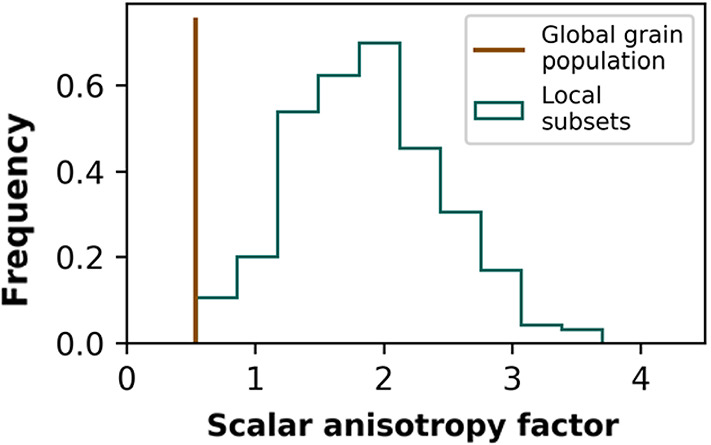
Distribution of local anisotropy computed on 300 local subsets of grains in the pluviated sample, compared with the global sample anisotropy. Each subset consists of a randomly chosen grain along with its 20 nearest neighbors

Two different depositional processes may explain the differences in preferred spatial orientations of the sand grains. The horizontal preferred orientation of the long axis of the pluviated sand grains is consistent with existing studies of laboratory sands using photographic [15] or radiographic [33] methods, which find that grains align normal to the direction of pouring. However, we find that the pluviated sand grains are not distributed symmetrically around the vertical axis (Fig. 4). We propose that the orientation of newly deposited grains influences the orientation of subsequently deposited grains. Thus, if enough of the initially deposited grains randomly align azimuthally, the subsequently deposited grains follow suit. An analysis of local orientations in the pluviated sample supports this idea (Fig. 7). Subsets of neighboring grains are more anisotropic than the global grain assembly, suggesting that pluviated grains’ orientations may be influenced by their previously deposited neighbors.

As sample collection does not preserve the core’s azimuthal orientation, the preferred azimuth direction of the naturally-deposited sands is unknown. However, grains generally develop preferred orientations parallel to the flow direction [17]. The large range of the preferred azimuth (>90 degrees) could arise from different swash and backwash flow directions. The preferred elevation of the naturally-deposited sands is not horizontal, even though flow was horizontal (beach slope was <2 degrees). Instead, the naturally-deposited grains have elevations between 0 and 30 degrees from the horizontal (Figs. 4, 5). We propose that beach sands are originally deposited with a horizontal orientation, but this horizontality is quickly disturbed by swash and backwash. The preferred orientation is reminiscent of the imbrication seen in larger grains in deposits from rivers [34, 35], submarine sediment flows that form turbidites [36, 37], and some volcanic particle-laden flows [38], though here preserved in sand-size particles. Imbrication is attributed to bedload transport wherein particles roll over a surface [39]. In contrast, pluviation does not introduce repeated disturbances, which can explain how a strong fabric anisotropy (i.e., preferred horizontality) is retained in the pluviated sand grains.

## Conclusion

Laboratory sands are often used in experimental studies of sand behavior. However, difficulty lies in extrapolating results to naturally-deposited sands, because different depositional processes may impart distinct microstructures, which influence macroscale behavior. We use x-ray computed microtomography to quantify key microstructural parameters in pluviated and naturally-deposited sands. Naturally-deposited sands have a lower frequency of coordination numbers less than 6 and greater than 16. The pluviated sands exhibit a strong horizontal preferred orientation, while the naturally-deposited sands exhibit an imbricated preferred orientation. We propose that flowing water at beaches (e.g., waves, swash, and backwash) remobilizes and reorients sand grains, resulting in fewer unstable grain configurations and a lower degree of fabric anisotropy in naturally-deposited sands than their pluviated counterparts.

Studies that investigate depositional method, including the present study, commonly analyze a small number of samples [40–42]. Thus, future experimental studies on depositional method will benefit from testing multiple samples for each preparation method in order to establish reproducibility. Inclusion of such experimental studies will allow for depositional method, microstructure, and macroscale behavior to be explicitly quantified and linked.
